# Clinical Predictors of Longitudinal Respiratory Exacerbation Outcomes in Young Hospitalised Children

**DOI:** 10.1111/cea.70099

**Published:** 2025-06-22

**Authors:** Laura A. Coleman, Siew‐Kim Khoo, Kimberley Franks, Franciska Prastanti, Jonatan Leffler, Peter N. Le Souëf, Yuliya V. Karpievitch, David G. Hancock, Ingrid A. Laing

**Affiliations:** ^1^ Medical School (Paediatrics) University of Western Australia Crawley Australia; ^2^ Wal‐Yan Respiratory Research Centre The Kids Research Institute Australia Nedlands Australia; ^3^ School of Biomedical Sciences University of Western Australia Crawley Australia; ^4^ School of Population Health Curtin University Perth Australia; ^5^ Department of Respiratory and Sleep Medicine Perth Children's Hospital Nedlands Australia

**Keywords:** acute asthma, asthma exacerbation, childhood, risk factors, wheezing

**Summary**

- Several clinical factors were associated with future respiratory exacerbation risk in the MAVRIC cohort.
- Multivariable models showed poor predictive performance with no combination of factors reliably predicting long‐term outcomes.

To The Editor,

Respiratory infection and wheezing illness are leading causes of hospitalisation in childhood, placing a significant burden on families and healthcare systems. However, reliably distinguishing children at risk of developing persistent disease from those likely to outgrow their symptoms remains a clinical challenge [1]. Earlier identification would allow clinicians to focus care and resources on those most likely to benefit from long‐term management, while reducing anxiety and uncertainty about the future for families [2].

Although multiple studies have linked asthma and wheezing outcomes to clinical factors and investigation results, inconsistent findings among studies and the inherent heterogeneity of paediatric respiratory disease have limited translation into accurate risk prediction tools [3]. In this study, we used longitudinal data from the Mechanisms of Acute Viral Respiratory Infection in Children (MAVRIC) cohort to examine the association between well‐defined clinical factors and future respiratory exacerbation risk.

Children were recruited upon presentation to Perth Children's Hospital emergency department (previously Princess Margaret Hospital) with an acute lower respiratory tract illness, particularly wheeze and asthma, between 2006 and 2021. The subset of MAVRIC participants aged 0–5 years with complete data were included (*N* = 211). At recruitment, children completed a skin prick test, blood tests and viral/bacterial nasal swabs, while parents completed a detailed health questionnaire. Longitudinal respiratory‐related hospital presentations from birth until study completion were obtained from Western Australia's statewide public database. Time to next respiratory hospital exacerbation was defined as the time between recruitment and the next respiratory hospital presentation. Subsequent hospital exacerbations were defined as the total number of respiratory presentations from recruitment until study completion. Longitudinal exacerbation phenotypes were defined based on all hospital presentations from birth to the end of each participant's observation period and categorised into (1) ‘few’, (2) ‘multiple’ or (3) ‘persistent’ exacerbations, as previously described [4].

The median age of participants at recruitment was 2.71 years (range 0.08–4.96 years), with a male predominance (67%) and frequent atopy (79%) (Table 1). Participants were followed up for a median of 6.30 years (range 0.41–13.23 years). At least one subsequent hospital exacerbation was observed in 137 (65%) participants, at a median of 143 days (range 1–3113 days), with 107 (51%) re‐presenting within the first year. The number of subsequent exacerbations varied widely, with a median of 1 per participant (range 0–22). In total, 23 (13%) were classified with a persistent exacerbation phenotype, 58 (33%) as multiple and 94 (54%) as few, with 36 who could not be classified [4].

**TABLE 1:** Association between clinical characteristics and time until the next hospital respiratory exacerbation (Cox Proportional Hazards), total number of hospital respiratory exacerbations post‐recruitment (generalised linear model with negative binomial distribution and an offset for duration of follow‐up) and longitudinal hospital respiratory exacerbation phenotype (ordinal logistic regression).

Variable	Cohort distribution	Time to next exacerbation (Cox Proportional Hazards)		Total post‐recruitment exacerbations (generalised linear model)		Exacerbation phenotype (ordinal logistic regression)	
		Univariable hazard ratio (95% CI)	Multivariable hazard ratio (95% CI)	Univariable odds ratio (95% CI)	Multivariable odds ratio (95% CI)	Univariable odds ratio (95% CI)	Multivariable odds ratio (95% CI)
Age at recruitment (median, range)	2.71 years (0.08–4.96)	1.018 (0.901–1.150)		1.212 (1.007–1.457)*		1.140 (0.917–1.422)	
Female sex (*N*, %)	70 (33%)	0.898 (0.628–1.286)		0.688 (0.434–1.107)		0.670 (0.357–1.237)	
Number of other children in household (median, range)	2 (0–8)	0.867 (0.614–1.224)	**0.843** (0.723–0.984)*	0.890 (0.766–1.041)	0.848 (0.736–0.982)*	0.833 (0.650–1.047)	0.805 (0.613–1.036)
Family history of respiratory disease (*N*, %)	65 (31%)	0.948 (0.658–1.367)		1.173 (0.736–1.904)		1.699 (0.914–3.158)	1.843 (0.946–3.614)
Season at recruitment (*N*, %)	Spring/Summer: 48 (23%) Winter/Autumn: 163 (77%)	0.904 (0.611–1.338)		0.903 (0.526–1.502)		0.796 (0.396–1.625)	
Atopy (*N*, %)	166 (79%)	1.476 (0.950–2.295)		1.857 (1.070–3.125)*		2.061 (1.029–4.304)*	
Number of previous hospital respiratory exacerbations (median, range)	1 (0–12)	1.170 (1.107–1.237)***	1.160 (1.094–1.229)***	1.308 (1.181–1.464)***	1.285 (1.161–1.434)***	N/A	
RV‐A positive at recruitment (*N*, %)	43 (20%)	1.279 (0.859–1.909)		0.785 (0.463–1.383)		0.938 (0.460–1.870)	
RV‐B positive at recruitment (*N*, %)	3 (1%)	0.350 (0.049–2.504)		0.227 (0.033–2.564)		0.513 (0.024–4.517)	
RV‐C positive at recruitment (*N*, %)	92 (44%)	1.301 (0.930–1.821)		1.695 (1.103–2.617)*		2.504 (1.397–4.537)**	1.806 (0.971–3.376)
RSV positive at recruitment (*N*, %)	40 (19%)	0.576 (0.362–0.918)*	0.596 (0.364–0.977)*	0.315 (0.175–0.578)***	0.417 (0.239–0.738)**	0.263 (0.106–0.590)**	0.337 (0.131–0.792)*
Eosinophils above upper end of normal (*N*, %)	78 (37)	1.532 (1.089–2.156)*		1.366 (0.877–2.153)		1.729 (0.953–3.144)	
Neutrophils above upper end of normal (*N*, %)	82 (39)	1.337 (0.953–1.878)		1.347 (0.868–2.110)		1.579 (0.873–2.858)	
Lymphocytes below lower end of normal (*N*, %)	70 (33)	0.782 (0.542–1.128)	0.640 (0.437–0.936)*	1.112 (0.706–1.778)		1.128 (0.609–2.073)	

*Note:* Variables were first assessed one at a time in univariable models. A multivariable model was then constructed using step‐wise regression to select variables by optimising the Akaike information criterion. *, *p* < 0.05; **, *p* < 0.01; ***, *p* < 0.001. Bold values indicate a statistically significant association between the variable and outcome.

Abbreviation: CI = confidence interval.

In univariable and multivariable models, other children in the household, rhinovirus‐C (RV‐C) infection, RSV infection, eosinophil counts and lymphocyte counts were significantly associated with at least one outcome (Table 1). The predictive power of the multivariable models was modest–poor for time to next exacerbation (Area Under the Curve (AUC) 0.74), number of subsequent exacerbations (AUC 0.48) and longitudinal exacerbation phenotype (AUC 0.55).

The choice of clinical endpoint is critical in paediatric respiratory research and is a major contributor to the observed heterogeneity across studies [5]. To overcome these limitations, we chose to analyse three clinically relevant outcome measures, as no single factor fully captures the complexity of longitudinal respiratory disease morbidity in early childhood [6]. Consistent with this heterogeneity, only RSV detection demonstrated a consistent negative association with all three outcomes (*p* < 0.05), although most other assessed factors generally demonstrated a consistent direction of association.

Our observed associations between respiratory viral pathogen (i.e., RV‐C/RSV) at initial presentation and subsequent respiratory morbidity align with existing literature [7, 8]. While positive associations have been reported for both rhinovirus and RSV [9], rhinovirus infection (particularly RV‐C), has been consistently identified as a central player [7, 8]. Therefore, the negative association between RSV positivity and three longitudinal outcomes in our study, may better reflect an absence of RV‐C infection, as co‐infections were rare in our cohort. Overall, our findings reinforce the role of specific viral species in shaping long‐term respiratory outcomes, although underlying biological mechanisms remain unclear.

Previous risk prediction tools have generally demonstrated limited performance and reproducibility, likely reflective of both the complexity of paediatric wheezing disease and differences in clinical endpoints between studies [3]. Despite our study assessing well‐characterised clinical factors with proven associations, our multivariable models lacked robust predictive accuracy. Our results in the context of the published literature [3] suggest that existing clinical markers are insufficient to accurately predict future disease risk.

The MAVRIC cohort represents a large, real‐world population of children followed over a long follow‐up period with data on multiple clinically relevant endpoints that allowed us to comprehensively assess a spectrum of future respiratory risk; however, there are some limitations to consider. Longitudinal outcome data was based on public health record data, which would miss private hospital presentations (rare in Western Australia) and minor community exacerbations. Classification in real‐world cohorts can also change as more data become available over time. In addition, a subset of children in the cohort displayed intermediate phenotypes and could therefore not be assigned a persistent/multiple/few longitudinal exacerbation phenotype and were removed from this analysis. However, this classification difficulty also mirrors real‐world variability in respiratory trajectories and clinical phenotyping challenges in early childhood. While we assessed well‐characterised clinical factors, additional variables such as parental smoking were not explored.

In summary, we identified consistent associations between clinical factors and longitudinal respiratory outcomes, but these factors showed limited predictive accuracy in multivariable models. Our data highlights the need to develop novel biomarkers that correlate with treatable traits to stratify risk and inform treatment/surveillance decisions.

## Author Contributions

**Laura A. Coleman:** formal analysis, methodology, software, visualization, writing – original draft. **Siew‐Kim Khoo:** data curation, investigation, project administration. **Kimberley Franks:** data curation, investigation. **Franciska Prastanti:** data curation, investigation. **Jonatan Leffler:** supervision, writing – review and editing. **Peter N. Le Souëf:** conceptualization, funding acquisition, methodology, project administration, supervision, writing – review and editing. **Yuliya V. Karpievitch:** methodology, supervision, writing – review and editing. **David G. Hancock:** methodology, supervision, writing – review and editing. **Ingrid A. Laing:** conceptualization, funding acquisition, methodology, project administration, supervision, writing – review and editing.

## Ethics Statement

This study was granted ethics approval from Princess Margaret Hospital for Children (Ethics #1761EP) and Perth Children's Hospital (RGS #2369) and informed consent was obtained from at least one parent/guardian prior to recruitment.

## Conflicts of Interest

The authors declare no conflicts of interest.

## Data Availability

The study data are available on request from the corresponding author. The data are not publicly available due to privacy or ethical restrictions. Full methods and results can be found at: https://doi.org/10.5281/zenodo.15393369 (Supporting Information).
